# Identification of Bacterial Profiles and Their Interactions with Selected Quality, Oxidative, and Immunological Parameters of Turkey Semen

**DOI:** 10.3390/ani11061771

**Published:** 2021-06-14

**Authors:** Michal Lenický, Tomáš Slanina, Miroslava Kačániová, Lucia Galovičová, Michaela Petrovičová, Michal Ďuračka, Filip Benko, Ján Kováč, Eva Tvrdá

**Affiliations:** 1 Department of Animal Physiology, Faculty of Biotechnology and Food Sciences, Slovak University of Agriculture, Tr. A. Hlinku 2, 94976 Nitra, Slovakia; michallenicky2@gmail.com (M.L.); tomas.slanina@uniag.sk (T.S.); m.petrovicova14@gmail.com (M.P.); michaelduracka@gmail.com (M.Ď.); filip.benko276@gmail.com (F.B.); jan.johnny.kovac@gmail.com (J.K.); 2 Department of Fruit Science, Viticulture and Enology, Faculty of Horticulture and Landscape Engineering, Slovak University of Agriculture, Tr. A. Hlinku 2, 94976 Nitra, Slovakia; kacaniova.miroslava@gmail.com (M.K.); l.galovicova95@gmail.com (L.G.); 3 Department of Bioenergetics, Food Analysis and Microbiology, Institute of Food Technology and Nutrition, University of Rzeszow, Cwiklinskiej 1, 35-601 Rzeszow, Poland; 4 Department of Neuroscience, Second Faculty of Medicine (2. LF UK), V Úvalu 84, 150 06 Prague, Czech Republic

**Keywords:** turkey, semen quality, bacteriospermia, bacterial profiles

## Abstract

**Simple Summary:**

Overbreeding—and its associated increase in the chest areas of turkeys—has led to the loss of their natural ability to reproduce. Therefore, commercial production of turkey meat relies on artificial insemination. However, along with the physiology of the genital tract of turkeys, there is high potential for bacterial contamination of ejaculates. These bacteria may affect crucial semen quality parameters required for successful fertilization. As such, it is important to pay close attention to the bacteria present in turkey ejaculates and possible solutions to eliminate their adverse effects on avian spermatozoa.

**Abstract:**

This study focused on the identification of naturally occurring bacteria in the reproductive fluid and impact on the quality of ejaculates obtained from the turkey breed British United Turkeys (BUT) Big 6 (n = 60). We determined possible relationships between the bacterial load and advanced sperm quality parameters that are important for effective artificial insemination and high fertility, as well as the concentration of selected antimicrobial proteins and pro-inflammatory markers of turkey semen. Sperm motility was assessed with computer-assisted sperm analysis (CASA), while the membrane and acrosome integrity were examined with smearing and staining methods. Reactive oxygen species (ROS) generation was quantified via luminometry, sperm DNA fragmentation was evaluated using the TUNEL assay, and the JC-1 assay was applied to evaluate the mitochondrial membrane potential. Cell lysates were prepared to investigate the extent of lipid and protein oxidation. Furthermore, levels of interleukins 1 and 6 (IL-1, IL-6), C-reactive protein, cathelicidin, and β-defensin were quantified in the seminal plasma using the ELISA method. The most dominant species identified by the matrix-assisted laser desorption/ionization time-of-flight (MALDI-TOF) mass spectrometry was *Escherichia coli*, *Proteus mirabilis*, *Staphylococcus lentus,* and *Citrobacter braakii*. The bacterial load had a negative effect on the sperm motility (*p* < 0.001), as well as membrane (*p* < 0.05) and acrosome integrity (*p* < 0.01). A strong positive relationship between the bacterial load and DNA fragmentation (*p* < 0.001) was detected as well. Positive associations were recorded between the increasing presence of bacteria, ROS overgeneration (*p* < 0.001), and a subsequent oxidative damage to the proteins (*p* < 0.001) and lipids (*p* < 0.01). It was revealed that the antimicrobial peptides β-defensin (*p* < 0.001) and cathelicidin (*p* < 0.001) had a positive relationship with the motility. In contrast, pro-inflammatory markers, such as IL-1 (*p* < 0.001) and IL-6 (*p* < 0.001), had a negative impact on the motion behavior of turkey spermatozoa. Our results suggest that the semen quality may be notably affected by the bacterial quantity as well as quality. It seems that bacteriospermia is associated with inflammatory processes, oxidative stress, sperm structural deterioration, and a subsequent risk for a failed artificial insemination in turkey breeding.

## 1. Introduction

The global poultry industry has made enormous progress over the last three decades, particularly in the field of meat and egg production [1]. Massive overbreeding of turkeys, in favor of, for example, muscle incrementation in the chest area, has caused significant differences between males and female turkeys. This disparity has led to the loss of the turkey’s natural ability to reproduce; this is due to the male’s inability to physically approach the female’s cloaca in order for fertilization to transpire. While wild species have retained this capacity, commercial turkey production relies exclusively on artificial insemination for the selection of genetic information, encoding a broad breast as desirable for the meat industry [2,3].

The reproductive system of birds is unique in its anatomy [3], providing a great precondition for possible bacterial contamination of semen, which is a main factor influencing the success of artificial insemination. Specifically, semen passing through the *vas deferens* into the cloaca is at high risk of being infested with fecal bacteria originating from the gastrointestinal tract [2,4]. Sperm agglutination, morphological abnormalities, and DNA damage of male gametes, seminal oxidative stress, and a subsequent endangered fertility in humans, bulls, rabbits, and boars, have been often associated with bacterial infections of the urogenital tract [5,6,7,8]. The immune system plays an important role in preventing reproductive tract infections. A key component of the innate immune system of birds encompasses a group of antimicrobial peptides, such as β-defensins and cathelicidins. In addition to their antibacterial properties, these peptides may also regulate the production of cytokines and, thus, selectively support the host’s immune responses [9]. 

From a producer’s perspective, bacteria responsible for urogenital infections in males may be easily transmitted into females and, thus, affect production either indirectly (by potentially causing secondary infections to the hens) or directly (by contributing to a decline in the frequency of laying, as well as higher embryo mortality) [10]. Moreover, infections caused by *Salmonella*, *Clostridium* or the *Enterobacteriaceae* family may have a negative impact, not only on the bird’s welfare, but also on human health if contaminated products are consumed [10,11]. Subsequently, a potential horizontal or vertical transmission of bacteria in poultry production as a major cause of morbidity and mortality of the animals carries a heavy economic burden, which has been estimated to account for as much as 95.61% of the total economic loss that occurs in the commercial poultry industry [12,13,14,15].

Several strategies have been employed for the management of bacterial transmission in poultry production, including modern flock management practices, vaccinations against selected pathogenic bacteria, breeding programs to increase the resistance of animals against bacterial infections, sanitation programs, the use of an array of disinfection agents, as well as a high level of biosecurity [16,17]. From a historical perspective, antibiotics have become a preferred option for disease prevention and/or treatment in poultry, primarily because of their availability and cost-effectiveness. Nevertheless, recent research has revealed that, particularly *Escherichia coli*, which has been frequently detected in cloacal swabs from avian species, presents with a substantial resistance to tetracycline, ampicillin, streptomycin, ciprofloxacin, or erythromycin [18,19]. Such bacterial drug resistance has become a serious issue that may endanger public health. Another problem may lie in the subsequent escape of bacteria into the ecosystem and a possible horizontal transfer of resistance genes to other bacteria [19]. It is for this reason that the use of antibiotics in diluents and extenders suitable for poultry semen is nowadays strictly controlled [20]. 

As such, it is extremely important to pay close attention to the bacterial profiles of ejaculates with a special regard to the management or prevention of bacteriospermia in the future. While a microbiological analysis of semen is not a compulsory step within a routine protocol for artificial insemination in turkeys, we believe that the identification of the bacterial profiles may significantly help to select ejaculates of the best quality and, thus, decide on their use for maximum efficiency and sustainability of assisted reproduction. As opposed to traditional microbiological protocols based on morphological, biochemical,, and serological tests, our experimental approach takes advantage of the matrix-assisted laser desorption/ionization time-of-flight mass spectrometry (MALDI-TOF MS), which has emerged as a fast, reliable, and straight-forward technique to provide information on bacterial profiles of complex biological samples [21]. Moreover, it has already been successfully introduced to study stallion [22], rabbit [7], boar [8], and bovine semen [6].

The aim of our study was to describe bacterial profiles of turkey semen and to assess whether this may affect the sperm structural integrity and functional activity. Furthermore, we investigated the possible involvement of oxidative stress and selected immunological markers related to antibacterial protection in the evolution of bacteriospermia in turkeys.

## 2. Materials and Methods

### 2.1. Animals and Sample Collection

Semen samples for this study were obtained from the company Branko Nitra, a.s. (Nitra, Slovakia), which specializes in turkey production. The entire breeding process was subject to a strict veterinary control and complied with the regulations valid within the EU ISO 9001: 2000. Ejaculates were collected by massaging the cloaca of 60 sexually mature males from the Big 6 line (British United Turkey, Ltd.; Nitra-Dolné Krškany, Slovakia). To avoid possible external contamination, sterile collection syringes were used, and single-use gloves were changed between each animal. Prior to the collection, the animals were allowed to defecate and, subsequently, their cloacae were washed with soapy water. After collection, the ejaculates were transported to the laboratory in a thermodynamically sealed system. The animals were carefully handled in accordance with ethical guidelines of the Slovak Animal Protection Regulation RD 377/12, conforming to European Union Regulation 2010/63.

Each sample was diluted in PBS (phosphate buffered saline; Sigma-Aldrich, St. Louis, MO, USA) using a ratio of 1:100, and subjected to the assessment of the sperm motility, reactive oxygen species (ROS) production, mitochondrial membrane potential, membrane, and acrosome integrity.

A portion of each ejaculate was centrifuged (Hettich Rotina 420, Tuttlingen, Germany) at 2500 RPM for 5 min to separate the cells from the seminal plasma. Seminal plasma aliquots were stored at −80 °C for a subsequent analysis of the total antioxidant status (TAS), interleukin 1 (IL-1), interleukin 6 (IL-6), C-reactive protein (CRP), cathelicidin (CATH) and β-defensin (DEF). The cells were lysed with the RIPA buffer (Sigma-Aldrich, St. Louis, MO, USA) containing a protease inhibitor cocktail (Sigma-Aldrich, St. Louis, MO, USA) and disrupted using a sonicator (28 kHz) for 45 s. During sonication, the samples were placed on ice. The last step consisted of the centrifugation of the resulting mixture (10 min, 5000 RPM, 4 °C) and sample purification. The resulting cell lysates were stored at −80 °C for the determination of lipid peroxidation (LPO) and oxidative damage to the proteins.

### 2.2. Sperm Motility

The motility was analyzed using the CASA (computer-assisted sperm analysis) system (version 14.0 TOX IVOS II, Hamilton-Thorne Biosciences, Beverly, CA, USA). Seven µL of the diluted sample were transferred to a Makler counting chamber (10 µm depth; Sefi Medical Instruments, Haifa, Israel), which was placed into a pre-heated plate set at 37 °C. The system evaluated the turkey sperm motion by automatically scanning 10 different microscopic fields through the chamber. Sperm motion was expressed as a percentage (%) of cells with a motility rate >5 μm/s.

### 2.3. Membrane Integrity

Membrane integrity was determined using a colorimetric approach. Two μL of the diluted sample were placed on a microscopic slide using a pipette. Subsequently, 4 μL of the eosin dye (Eosin Y; Sigma-Aldrich, St. Louis, MO, USA) were added on the slide, followed by 4 μL of the nigrosin contrast dye (Sigma-Aldrich, St. Louis, MO, USA). With the help of a second slide, a smear was created and allowed to dry at room temperature. The slides were evaluated on an inverted light microscope (Leica DM IL LED, Leica Microsystems, Wetzlar, Germany). Three hundred cells were counted diagonally from the upper left corner to the lower right edge of the slide by one experienced observer, and the proportion of living cells was expressed in percentage (%) [23].

### 2.4. Acrosome Integrity

A microscopic methodology was used to determine the integrity of the acrosome [24]. Diluted samples (20 μL) were stained with 20 μL of a staining solution consisting of fast green (Sigma-Aldrich, St. Louis, MO, USA) and rose bengal (Sigma-Aldrich, St. Louis, MO, USA). The sample was allowed to incubate at room temperature for 70 s. After incubation, 10 μL of the mixture were smeared on a slide and dried at room temperature. The slides were evaluated by one experienced observer under an inverted light microscope (Leica DM IL LED, Wetzlar Germany) by counting 300 cells. Cells displaying an intact acrosome cap were expressed in percentage (%).

### 2.5. Quantification of Leukocytes

Quantification of leukocytes was performed with the Endtz test. The working solution (WS) consisted of 96% ethanol (Centralchem, Bratislava, Slovakia), benzidine (Sigma-Aldrich, St. Louis, MO, USA), sterile water, and 3% hydrogen peroxide (Sigma-Aldrich, St. Louis, MO, USA). Twenty µL of diluted specimen (1:100) were mixed with 40 µL of WS and left to incubate in the dark (5 min; room temperature). Stained round cells were counted using the Makler counting chamber under a bright-field microscope (×1000; Nikon ECLIPSE E100, Tokyo, Japan) [25]. The results are quoted as × 10^6^ white blood cells/mL.

### 2.6. Mitochondrial Membrane Potential (ΔΨm)

The mitochondrial membrane potential was determined using the JC-1 Mitochondrial Membrane Potential Assay kit (Cayman Chemical, Ann Arbor, MI, USA). The key component is the lipophilic, light-sensitive cationic dye JC-1 (5.5′,6.6′-tetrachloro-1,1′,3,3′-tetraethylbenzimidazolylcarbocyanine iodide), which was diluted in PBS shortly prior to the analysis. Hundred μL of the sample were stained with 5 μL of JC-1 working solution and incubated for 30 min at 37 °C. After incubation, the samples were centrifuged (5 min, 2100 RPM, 25 °C) and washed twice with a washing buffer provided by the commercial kit. Finally, the sample was transferred to a dark 96-chamber plate. The plate was analyzed using a combined GloMax-Multi+ spectro-fluoro-luminometer (Promega, Madison, WI, USA) [7]. The resulting ΔΨm is expressed as the ratio of JC-1 complexes to JC-1 monomers (green/red ratio).

### 2.7. TUNEL Assay

Sperm DNA fragmentation was evaluated using the APO-DIRECT^TM^ TUNEL assay kit (BD Biosciences; Franklin Lakes, NJ, USA). One million cells were collected from each sample, fixed in 4% paraformaldehyde (Centralchem, Bratislava, Slovakia), and incubated on ice for 1 h. Subsequently the cells were washed 3 times in PBS and stored in 70% ice-cold ethanol (Centralchem, Bratislava, Slovakia) overnight at −20 °C. Following storage, the cells were washed, labeled with the DNA labeling solution, rinsed, and centrifuged (5000 RPM, 5 min) twice. The pellet was subsequently incubated in propidium iodide/RNase staining buffer for 30 min in the dark. Following incubation, each sample was counterstained with DAPI (Sigma-Aldrich, St. Louis, MO, USA) and placed into a dark 96-well plate. Appropriate fluorescent signals were obtained using the Glomax Multi+ spectro-fluoro-luminometer [26]. The proportions of cells with fragmented DNA as well as necrotic cells are expressed in percentage (%).

### 2.8. ROS Production

To determine the quantity of ROS produced by spermatozoa, the chemiluminescent method was used, which may detect light emitted by a chemical reaction. The luminescent probe used in the experiment was luminol (5-amino-2,3-dihydro-1,4-phthalazinedione; Sigma-Aldrich, St. Louis, MO, USA). Samples (100 μL) were transferred into 96 chamber plates, which also contained the blank (100 μL PBS), negative control (100 μL PBS), and positive control [100 μL PBS, 12.5 μL 30% hydrogen peroxide (H_2_O_2_; 30%; 8.8 M; Sigma-Aldrich, St. Louis, MO, USA)]. Subsequently, 2.5 μL of luminol working solution were added to the sample, positive and negative control. The light signal was monitored in 15 consecutive one-minute-long cycles using the Glomax Multi+ spectro-fluoro-luminometer. The results are expressed as relative light units (RLU)/s/10^6^ spermatozoa [7].

### 2.9. Total Antioxidant Status

The total antioxidant status of the seminal plasma was determined using the improved chemiluminescence antioxidant assay, which utilizes horseradish peroxidase conjugate and luminol. Trolox (5–100 μmol/L; 6-hydroxy-2,5,7,8-tetramethylchroman-2-carboxylic acid; Sigma-Aldrich; St. Louis, MO, USA) served as the standard and a signal reagent, consisting of 0.1 mol/L Tris-HCl (Sigma-Aldrich; St. Louis, MO, USA), 12 mol/L H_2_O_2_ (Sigma-Aldrich; St. Louis, MO, USA), 41.8 mmol/L 4-iodophenol (Sigma-Aldrich; St. Louis, MO, USA); and 282.2 mmol/L luminol (Sigma-Aldrich; St. Louis, MO, USA) was employed to induce the chemiluminescent reaction. The light signal was processed on 96-well plates in 10 consecutive one-minute-long cycles using the Glomax Multi+ spectro-fluoro-luminometer. The results are expressed as μmol Trolox equivalent/g protein [26].

### 2.10. Enzyme-Linked Immunosorbent Assay (ELISA)

Selected cytokines (IL-1, IL-6), inflammatory proteins (CRP) and antimicrobial peptides (CATH, DEF) present in the seminal plasma were determined using commercially available ELISA kits (Chicken Cathelicidin Antimicrobial Peptide ELISA Kit; Avian Beta-Defensin ELISA Kit; Chicken C-reactive protein ELISA Kit; Chicken Interleukin 1 ELISA Kit; Chicken Interleukin 6 ELISA Kit; MyBioSource Inc., San Diego, CA, USA). All experimental quantifications were based on double-sandwich ELISA taking advantage of a primary pre-coated monoclonal antibody while the detecting antibody was a biotin-labeled polyclonal antibody. All ELISA protocols were performed as per instructions of the manufacturer and executed with the help of the Glomax Multi+ spectro-fluoro-luminometer at 450 nm.

### 2.11. Lipid Peroxidation

Thiobarbituric acid-reactive substances (TBARS) is a method to detect and quantify malondialdehyde (MDA) as a pre-dominant by-product of LPO. One hundred μL of the sperm lysate were processed with 100 μL 5% SDS (sodium dodecyl sulfate; Sigma-Aldrich, St. Louis, MO, USA) and 4 mL 0.53% thiobarbituric acid (Sigma-Aldrich, St. Louis, MO, USA) dissolved in 20% acetic acid (pH 3.5; Centralchem, Slovakia). For the reaction to proceed, the samples were heated in a water bath at 100 °C for 60 min. Following heating, immediate cooling of the mixture was required to stop the ongoing reaction, and therefore the samples were stored on ice for 10 min. The samples were then centrifuged at 3800 RPM for 10 min. The resulting supernatant was pipetted into a 96-well plate and assessed using the Glomax Multi+ spectro-fluoro-luminometer at a wavelength of 540 nm. MDA concentrations were calculated using a standardization curve constructed from pre-prepared MDA standards. The results are quoted as µmol MDA/g protein [26].

### 2.12. Protein Carbonyls

To assess the extent of protein oxidation, the quantity of protein carbonyls (PC) in the cell lysates was evaluated using a modified dinitrophenylhydrazine (DNPH) method introduced by Weber et al. [27]. Each sample was adjusted to contain 1 mg protein/1 mL, treated with 1 mL of trichloroacetic acid (TCA; Sigma-Aldrich, St. Louis, MO, USA) and stored for 10 min at 4 °C. After incubation, the samples were centrifuged (3000 RPM, 10 min) and the pellet was incubated for 60 min at 37 °C in the presence of 1 mL DNPH (Sigma-Aldrich, St. Louis, MO, USA). After incubation, 1 mL TCA was added again; the samples were then cooled down and centrifuged (3000 RPM, 5 min). The pellet was washed 3 times with 500 µL of a mixture of ethanol and ethyl acetate (1:1; Sigma-Aldrich, St. Louis, MO, USA). Finally, the pellet was resuspended in 1 mL of 6 M guanidine hydrochloride (Sigma-Aldrich, St. Louis, MO, USA). The resulting measurement for the determination of protein carbonyls was performed at 360 nm on a UV–VIS spectrophotometer (Cary Systems, Santa Clara, CA, USA). Oxidative damage to the proteins is expressed in nmol PC/mg protein.

### 2.13. Data Normalization

To normalize the collected data, it was necessary to determine the total protein concentration in each seminal plasma and sperm lysate specimen. Following the Biuret method, the proteins reacted with copper sulfate in an alkaline medium to produce a violet–blue color. The intensity of the color was directly proportional to the protein concentration in the sample. The protein concentration was determined using the commercial Total Protein kit (DiaSys, Holzheim, Germany) and the semi-automated Monza photometric analyzer (Randox Laboratories, Crumlin, UK) at 540 nm.

### 2.14. Bacteriological Identification

For the identification of the bacterial colonies and species in turkey semen, 100 µL of each sample were inoculated onto a sterile blood agar (BA, Blood Agar Base No. 2; Merck, Darmstadt, Germany) and tryptone soya agar (TSA, Soyabean Casein Digest Agar; Merck, Darmstadt, Germany). Subsequently, the plates were incubated under aerobic conditions at 36 ± 2 °C for 24 h. Following cultivation, the colonies were counted and transferred to a fresh TSA to obtain pure cultures, which were incubated again for 24 h at 37 ± 1 °C under aerobic conditions.

### 2.15. Bacteriological Analysis (MALDI-TOF MS Biotyper)

The purified bacterial cultures were subsequently identified by matrix assisted laser desorption/ionization time-of-flight (MALDI-TOF) Biotyper mass spectrometry (Brucker Daltonics, Bremen, Germany).

Using an inoculation loop, a small amount of a purified culture was mixed with 300 μL of distilled water. Subsequently, 900 μL, 99.8% ethanol (Centralchem, Bratislava, Slovakia) were added and the samples were centrifuged at 3200 RPM for 2 min at room temperature. The supernatant was discarded, and the pellet deposited on the bottom could dry freely. The pellet was then resuspended thoroughly by adding 30 μL 70% formic acid (Sigma-Aldrich, St. Louis, MO, USA) and the same amount of acetonitrile (Sigma-Aldrich, St. Louis, MO, USA). Once again, the samples were centrifuged at 3500 RPM at room temperature for 2 min. One μL of the supernatant was placed on a 96-point MALDI identification plate and allowed to dry freely.

Prior to the identification a working solution of MALDI matrix was prepared, containing acetonitrile, ultrapure water, and trifluoroacetic acid (Sigma-Aldrich, St. Louis, MO, USA) in a ratio of 20:19:1. A volume of 250 μL of the MALDI solution was mixed with a small amount of cinnamic acid powder (Sigma-Aldrich, St. Louis, MO, USA). The mixture was poured over the plate with the dried supernatant. Identification of the bacterial species was performed using the Microflex LT instrument equipped with the flexControl software version 3.4. The spectra measured by mass spectrometry were compared with the MALDI Biotyper Bruker Taxonomy database (Bruker Daltonics, Bremen, Germany) [7].

### 2.16. Antibiotic Resistance Testing

Bacterial species isolated from semen were tested for antibiotic resistance. The antimicrobial susceptibility test was performed with the disc diffusion method against (10 mg) cefepime (FEP), ertapenem (ETP), chloramphenicol (C), linezolid (LZD), norfloxacin (NOR), and tigecycline (TGC), as previously described by Kačániová et al. [28].

### 2.17. Statistical Analysis

GraphPad Prism statistical program (version 8.4.3 for Mac; GraphPad Software Incorporated, La Jolla, CA, USA) was used for the data analysis. General characteristics of all assessed quality markers are expressed as mean ± standard deviation (S.D.). All data were subjected to Pearson correlation analysis. The interpretation of the results was based on the value of the Pearson correlation coefficient (r): ±0.111–±0.333: weak correlation; ±0.334–±0.666: moderate correlation; ±0.667–±0.999: strong correlation. For a deeper analysis of the collected data, the samples were divided according to the sperm motility rates into excellent-quality group (EX; MOT > 70%; n = 22), good-quality group (GO; MOT > 50%; n = 20) and low-quality group (LO; MOT < 50%; n = 18). Differences between the individual qualitative groups were evaluated by one-way ANOVA and followed by the Tukey multiple comparison test. Levels of statistical significance for both statistical operations were set at: * *p* < 0.05; ** *p* < 0.01; *** *p* < 0.001; **** *p* < 0.0001.

## 3. Results

### 3.1. Microbial Analysis

**Figure 1:**
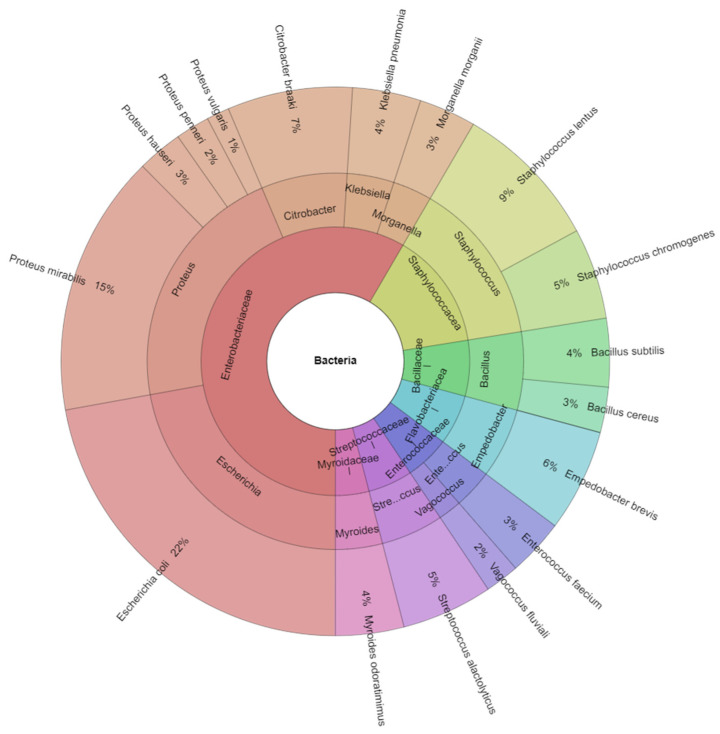
Krona chart of the bacteria represented by MALDI-TOF MS Biotyper recovered from turkey semen (outermost ring: species, middle ring: genus, innermost ring: family).

Using MALDI-TOF mass spectrometry, 7 families, 12 genera, and 17 bacterial species were identified in turkey ejaculates (Figure 1): *Bacillus cereus* (*B. cereus*), *Bacillus subtilis* (*B. subtilis*), *Citrobacter braakii* (*C. braakii*), *Empedobacter brevis* (*E. brevis*), *Enterococcus faecium* (*E. faecium*), *Escherichia coli* (*E. coli*), *Klebsiella pneumoniae* (*K. pneumoniae*), *Morganella morganii* (*M. morganii*), *Myroides odoratimimus* (*M. odoratimimus*), *Proteus hauseri* (*P. hauseri*), *Proteus mirabilis* (*P. mirabilis*), *Proteus penneri* (*P. penneri*), *Proteus vulgaris* (*P. vulgaris*), *Staphylococcus chromogenes* (*S. chromogenes*), *Staphylococcus lentus* (*S. lentus*). *Streptococcus alactolyticus* (*S. alactolyticus*), and *Vagococcus fluvialis* (*V. fluvialis*).

All bacterial isolates acquired from turkey ejaculates were tested for antimicrobial resistance (Table 1) against cefepime, ertapenem, imipenem, chloramphenicol, linezolid, norfloxacin, or tigecycline. The resulting inhibition zones were evaluated according to the European Committee on Antimicrobial Susceptibility Testing (EUCAST) recommendations. All *C. braakii*, *P. hauseri*, *P. mirabilis*, *P. penneri,* and *P. vulgaris* isolates (100%) were sensitive to the antibiotics selected for the respective testing. On the other hand, all *S. lentus* isolates (100%) were resistant to chloramphenicol, linezolid, and tigecycline. In the case of *E. faecium*, resistance was detected against imipenem, while ertapenem showed to be ineffective against *E. coli* and *V. fluvialis*. All tested isolates were sensitive to cefepime, norfloxacin, and linezolid.

### 3.2. Analysis of the Qualitative Parameters of Semen

The descriptive statistical data of all assessed semen parameters are summarized in Table 2.

The correlation analysis (Table 3) revealed a general negative impact of the bacterial load on the quality parameters of the ejaculates. We observed a strong negative correlation between MOT and the bacterial load (*p* < 0.001; Table 3). Simultaneously, strong positive correlations (*p* < 0.001) were detected between the concentration of leukocytes, ROS production, and the presence of bacteria in the ejaculates. In the meantime, ROS production was in a negative association with the motility (*p* < 0.001) as well as other structures susceptible to oxidative stress, such as the membrane (*p* < 0.01) and acrosome (*p* < 0.05). While the stability of the membranous structures was in a strong positive relationship with the motility (*p* < 0.001), it was strongly negatively correlated (*p* < 0.001) with MDA levels. Acrosome integrity was also affected by the bacterial load as uncovered by a strong negative correlation (*p* < 0.01).

The mitochondrial membrane potential exhibited a strong positive correlation (*p* < 0.001) with the sperm motility, while being in a significant negative association with the bacterial load (*p* < 0.01) and ROS production (*p* < 0.001). A strong positive relationship was observed between CFU and the amount of protein carbonyls (*p* < 0.001), and a disruption of the antioxidant homeostasis of the ejaculate (*p* < 0.05). At the same time, we recorded significant positive correlations between the bacterial load and MDA (*p* < 0.01) as well as DNA fragmentation (*p* < 0.001). Furthermore, a positive correlation was recorded between DNA damage, leukocyte concentration, ROS production and the amount of MDA (*p* < 0.01) resulting from LPO.

The concentration of cathelicidin and β-defensin as proteins with antimicrobial activities exhibited a positive association with the sperm motility (*p* < 0.001) and, conversely, negative correlations with CFU and the presence of leukocytes (*p* < 0.01). At the same time, we observed that both proteins were positively associated with the total antioxidant capacity (*p* < 0.001), in contrast to the pro-inflammatory markers. Both interleukins, as well as CRP, were significantly negatively correlated with the motility (*p* < 0.001 in case of IL-1 and IL-6; *p* < 0.01 with respect to CRP). Inversely, strong positive associations were found between all inflammatory markers and the amount of leukocytes (*p* < 0.01 with regard to IL-1 and IL-6; *p* < 0.05 in case of CRP).

For a better interpretation of the causality of the obtained data, we divided the samples into three groups based on their motility rates: excellent-quality (EX; MOT > 70%; n = 22), good-quality (GO; MOT > 50%; n = 20), and low-quality (LO; MOT < 50%; n = 18) (Table 4).

The sample distribution analysis confirmed a direct impact of the bacterial load on the motion behavior of turkey gametes as revealed by significant differences in the percentage of motile spermatozoa between all three quality groups (*p* < 0.001). At the same time, our results suggest that the higher the quality of the sample, the higher the content of antimicrobial proteins. Significant differences in the CATH and DEF concentration were observed between the EX and the GO group (*p* < 0.001), as well as between the EX and LO group (*p* < 0.0001). The concentration of leukocytes was the highest in the LO quality group, presenting with significant differences among all quality groups (*p* < 0.001). In contrast, we did not observe significant differences in the CRP amount across the groups, suggesting that CRP may be present in semen independently of the type or quantity of bacteria detected in the specimen.

The highest concentration of IL-1 as a pro-inflammatory marker was detected in the LO group, which was significantly higher in comparison to the EX (*p* < 0.001), as well as the GO group (*p* < 0.001). The same observation was recorded in the case of IL-6 (*p* < 0.001 in case of EX vs. LO; *p* < 0.01 with respect to EX vs. GO).

In the case of ROS production, we did not observe a significant difference between the samples of the EX and the GO group. However, the data analysis revealed a significantly increased ROS concentration in the LO group when compared to the EX (*p* < 0.001) and the GO (*p* < 0.001) group. Correspondingly, protein oxidation was the most extensive in the LO group and being significantly higher when compared to the EX (*p* < 0.001) and the Go (*p* < 0.01) group. At the same time, samples included the EX group exhibited a significantly lower MDA content in comparison to the LO quality samples (*p* < 0.001). Moreover, significant differences were observed between the EX group and the LO group in case of TAC (*p* < 0.05), sperm DNA fragmentation (*p* < 0.05) and the occurrence of PI-positive spermatozoa (*p* < 0.01).

## 4. Discussion

The commercial turkey industry is dependent on artificial insemination; however, if the procedure is executed with a compromised semen sample, this will have a negative impact on the efficiency of the breeding program [29]. It is well known that various saprophytic and commensal bacteria are present in the gastroenteric tract of birds. The *vas deferens* outlet is located in close proximity to the ureteral outlet leading to the cloaca, which is why semen proceeding from the *vas deferens* presents with a natural predisposition to a potential fecal contamination [30].

Based on our collected data, it may be hypothesized that the sample quality may be partially determined by the occurrence of native antibacterial proteins and pro-inflammatory molecules. Furthermore, semen quality may be significantly affected by the quantity and variability of bacterial species present in the sample. The highest bacterial load was detected in the samples included in the LO group. Moreover, samples of low quality presented not only with the highest content, but also with the highest variability of bacterial species. It is important to note that typical uropathogenic and coliform bacteria, such as *E. coli*, were identified in the LO group exclusively. In fact, the majority of these samples were contaminated with coliform bacteria.

Ahmed et al. [30] focused on the bacteriological analysis of semen from Vanaraja roosters. Their traditional microbiological evaluation showed that, similar to our data, each ejaculate was contaminated with at least one bacterium, with a predominant presence of *E. coli* and *Klebsiella*. On the other hand, *Kluyvera ascorbata*, *Salmonella enteritidis*, *Pseudomonas,* or *Serratia plymuthica* were not detected in our case. Moreover, *Campylobacter* as a common bacterial cause for forborne infections was isolated from semen of commercial turkeys by Cole et al. [31], which was not the case in our study. A conservative approach, based on the gram staining and biochemical assays, was selected by Gale and Brown [32], who studied the bacterial profiles of semen collected from the Small White turkey breed. Overall, the authors identified fewer species than we did, while the bacterial diversity varied from case to case. When compared to our results, other species were recorded in the above-mentioned study except for *E. coli*; however, more similarities were found at the genus level, with *Staphylococcus* being frequent in both cases. At the same time, the authors ran a test of accuracy by analyzing semen samples obtained aseptically from the *vas deferens* and found no bacteria. This observation confirms the hypothesis that the ejaculate is not contaminated with bacteria until it passes through the cloaca. Furthermore, the bacterial load may differ significantly among the avian species. While the average bacterial load in 1 mL of turkey ejaculate oscillates around 1.3 billion bacteria [31,32], in roosters, the average concentration of bacteria may reach up to 2.2 million bacteria per mL [18,30].

On the one hand, pathogenic bacteria, such as *Salmonella* and *Clostridium* often found in poultry production have adverse effects on the animal survival, fertility, hatchery, and human health if products from such birds are consumed [10,11]. On the other hand, *Lactobacillus* spp. or *Bifidobacterium* spp. have a demonstrably positive effect on the avian gastrointestinal tract, and may be used as probiotics for poultry, with positive effects on the sperm production and hatchability [33]. Summarizing our MALDI-TOF data with the currently available evidence, we speculate that bacteriospermia does not necessarily indicate infection, as about 70% of semen samples usually contain non-pathogenic bacteria from the front of the urethra [34]. However, several authors noted an immediate decline in the avian semen quality following exposure to intestinal bacteria [18,29]. Based on these observations, further in vitro studies investigating the individual contribution of the bacteria identified in our samples to the sperm behavior are highly needed.

The mechanisms of action by which bacteria cause damage to male gametes are diverse. *Escherichia*, *Staphylococcus*, *Bacillus* or *Enterococcus*, all of which have been identified in our samples, are able to adhere to the sperm surface and subsequently affect cell-to-cell interactions [29,35,36,37]. While *Staphylococci* are known to cause permanent sperm agglutination through the agglutination factor (SAF) or immobilization factor (SIF) [36], *E. coli* may attach itself to receptors on the acrosome or sperm flagellum, leading to an impaired motility and the onset of cell death [35]. Our assessment of the membrane and acrosome integrity may agree with Schulz et al. [35] and Haines et al. [37], indicating that particularly *E. coli* infestation of semen may cause the sperm flagellum to tear off, knot, or break, and lead to defects in the acrosome, middle part, and head, with negative consequences on the overall fertilization ability. In our case, *E. coli* was detected in 67% of low-quality semen samples, suggesting its direct involvement in a notably decreased sperm vitality found in this category. The exact molecular mechanism by which the *E. coli* achieves such structural damage has not yet been precisely defined; however, our data indicate a possible role of membrane peroxidation caused by ROS production either directly by the bacterium or by inflammatory responses to its presence in semen.

Another possible mechanism of bacteria-associated spermatotoxicity is related to the secretion of lipopolysaccharide (LPS) endotoxins, hemolysin, or peptidoglycan fragments [38]. Their presence may lead to the activation of the toll-like receptor (TLR), which in turn ignites a pro-inflammatory immune response, secretion of cytokines, and antibacterial peptides, such as β-defensin [38,39]. Complementary to a positive association between bacterial load and the occurrence of necrotic cells observed in our study, Fujita et al. [38] reported that spermatozoa carrying TLRs embedded in their membrane can recognize bacterial endotoxins and, thus, activate a reaction cascade that induces apoptosis or necrosis.

It was previously reported that the negative impact of bacteria potentially responsible for urogenital tract infections on routine sperm quality markers is often associated with supraphysiological levels of seminal leukocytes [40,41,42,43,44,45]. A positive correlation between the bacterial load and the occurrence of leukocytes recorded in our study agrees with previous observations in animals [44,45] as well as humans [41,42,43]. Besides phagocytosis, seminal leukocytes present with other properties to eliminate pathogens, such as ROS production and the formation of extracellular traps (ETs). While both events are inherently designed to offer protection to the sperm survival in a bacteria-infested environment, a critical moment may arise when the ETs start to have a negative effect on the sperm motility, particularly by elevating oxidative tension and by physically trapping the male gametes. While this phenomenon has been observed in humans [46,47], cattle [44], and equines [45], mutual associations between the leukocyte concentration, ROS amounts, and the impairment of the sperm motility may be indicative of the assumption that ETs may emerge in bacteriospermic avian semen as well. As such, the involvement of leukocytes in the sperm immobilization, as a result of bacterial contamination of semen, is an intriguing area worth of further investigation.

Bacteriospermia has been frequently associated with a significant increase in sperm DNA fragmentation [6,7,48,49]. While an increased sperm DNA damage may be related to the actual presence of bacteria in the ejaculate, the degree of DNA fragmentation depends on numerous factors such as the concentration of the bacteria or their growth rate. An increased percentage of spermatozoa with fragmented DNA in the GO and LO groups might stem from previous hypotheses associated with oxidative insults to the DNA molecule [5,50]. These findings are also supported by strong associations of DNA damage with the presence of leukocytes, as well as the amounts of ROS, MDA, IL-6, and CFU as observed in our study. Furthermore, the release of bacterial endotoxins has also been directly associated with the apoptotic or necrotic process, which is accompanied by the loss of DNA integrity [49]. Similar to our results, semen samples infected particularly with *S. aureus*, *S. epidermis*, *S. haemolyticus*, *E. coli,* and *Enterococcus faecalis* exhibited high levels of DNA fragmentation and cell death, accompanied by a decreased motility and the onset of sperm membrane disintegration [48].

Inflammatory processes caused by pathogenic bacterial strains are accompanied by an outburst of ROS. The resulting oxidative imbalance is aggravated by the activation of the immune response to the presence of bacteria, generating even higher levels of ROS because of their inherent antibacterial properties. This accumulation of free radicals may ultimately cause a more profound damage to the functional activity of spermatozoa and potentially initiate cell death [5,7,50,51]. Based on our data, we propose the following sequence of events as a consequence of ROS overproduction associated with bacteriospermia: the primary target for seminal ROS are the membranous structures of spermatozoa, as these contains a large proportion of polyunsaturated fatty acids [52]. Subsequent oxidative insults will lead to irreversible changes in the membrane fluidity and permeability, as observed in a significant rise of the MDA levels accompanied by the loss of membrane integrity. Membrane destabilization will then result in protein inactivation and an increased risk for the disruption of a delicate internal milieu of the male gamete, which will be translated into the loss of motility or cell death [50,51], as indicated from our correlation analysis. We also agree with previous studies revealing that supraphysiological levels of ROS have been associated with mitochondrial ruptures and failure to effectively produce energy to sustain the sperm movement [26,52]. In summary, our data, along with evidence gathered from earlier reports, suggest that ROS production coupled with a deficient antioxidant protection may be prime catalysts responsible for the sperm dysfunction in semen infested with bacteria [7,50,51,52].

The first line of defense against pathogens represents the innate immune system, which plays an important role in the coordination of an appropriate biological response of the organism towards any intruders [53]. Our analysis of the seminal plasma focused on specific proteins associated with the activity of the immune system, which may play an important role in the immune activation, antigen presentation, and migration of white blood cells [53,54]. While it has been revealed that exposure of spermatozoa to LPS increases the expression levels of β-defensin [9,39], in our case, the molecule was found to be in a negative association with the quantity of bacteria present in semen. We speculate that once a critical threshold for the bacterial load is trespassed, β-defensin is not able to fully prevent or counteract damage to male gametes because of severe bacteriospermia. This assumption may be supported by relatively high levels of β-defensin in the semen samples of the EX group where the molecule was able to maintain the sperm vitality despite the presence of bacteria. Cathelicidin, similarly to β-defensin, has a strong antimicrobial activity against various fungi, bacteria, and viruses even at micromolar concentrations, which is why it may be included in the body’s natural defense system [53]. Corresponding to our collected data, a relationship between an increased susceptibility to infections and a decreased expression of cathelicidin has been reported in several studies [55,56]. Moreover, research in chickens has shown significant changes to the plasma cathelicidin following in vitro infection with bacterial endotoxins, suggesting that the molecule could act as a suitable marker for a fast detection of bacteriospermia in the breeding practice [57].

Immunocompetent cells in the male urogenital tract release various cytokines during bacterial infestation, which will mediate the course of the host’s innate immune response to fight infection [53,57]. These molecules also play an important role in cell-to-cell communication, while being able to modulate the pro-oxidant machinery during inflammation [53]. Elevated levels of the pro-inflammatory cytokine IL-6 and oxidative stress may play a pivotal role in the pathophysiology of infertility [5,58,59]. As reported by Cavaillon et al. [52], a significantly increased IL-6 secretion was observed following exposure of spermatozoa to bacterial LPS. These findings also correlate with our study where the concentration of IL-6 was higher in the samples containing more bacterial colonies. We observed the same trend for IL-1. The most plausible explanation for increased amounts of interleukins in semen samples of the GO and LO group may partially lie in an active recognition of bacterial proteins, which results in the activation of the sperm TLRs. This process initiates a series of reactions that leads to the cleavage of caspase 1, essential for IL-1 secretion [60]. Moreover, Fraczek et al. [5,50] indicated that pro-inflammatory cytokines promote ROS production to a level at which significant peroxidative damage occurs to the sperm membrane. Thus, cytokines alone increase the sperm sensitivity to oxidative stress while further ROS generation leads to a more severe deterioration of the male gamete. Additional research suggests that inflammatory mediators elicited by the immune response may be a direct cause of DNA fragmentation in spermatozoa [61]. This hypothesis was reinforced by our observation that increased oxidative tension in semen was in a direct association with pro-inflammatory cytokines, an increased LPO in the sperm membrane, and DNA disintegration resulting from the violation of the membrane integrity. While Martínez et al. [62] observed no effects of IL-6 on the lipid peroxidation in human spermatozoa, Eldamnhoury et al. [40] demonstrated that immune processes mediated by IL-1 and IL-6 were accompanied by a decreased progressive motility in infertile subjects. Meanwhile, Hagan et al. [41] noted a significant association between both IL-1β and IL-6 secretion and TLR expression on the sperm surface, confirming the importance of the receptor in inducing a defense response against the bacteria present in semen.

Levels of C-reactive protein (CRP) are often used as a diagnostic marker for bacterial infection commonly used in practice [63]. As tests for CRP are readily available and easy to use, we suggest their use for a fast screening of a potential bacterial contamination of semen, since, complementary to our findings, Leisegang et al. [64] reported that CRP was in a negative association with the sperm motility and vitality, while its levels were also positively correlated with seminal cytokines, suggesting a systemic inflammation with a direct negative impact on normal reproduction.

From the breeding point of view, it is desirable to eliminate the negative effects of the bacteria present in ejaculates, either by using suitable extenders or by a physical removal of bacteria during semen processing. At first glance, the simplest solution may lie in the supplementation of antibiotics. Penicillin was first antibiotic used in livestock production in the late 1940s, however its widespread administration has led to the occurrence of resistant bacteria, such as *E. coli* [18,19], or the methicillin-resistant *Staphylococcus aureus* colonial complex, formerly known as MRSA CC398 [65]. It is for this reason that the use of antibiotics is under a strict scrutiny in an intensive breeding process [16,17,19,20].

As such, more attention is devoted to the search for new substances with antimicrobial properties that could act as a suitable substitution for conventional antibiotics. A promising alternative could lie in the supplementation of antimicrobial peptides that are part of the innate immune system. The use of such molecules presents with a significantly lower risk for resistance development since these induce bacterial cell lysis in a non-specific manner [9,60].

Another possibility represents the use of various natural bioactive compounds such as resveratrol, quercetin and curcumin, or plant extracts and essential oils [7,28]. According to Schulze et al. [66], bacteria producing antimicrobial molecules themselves also have a promise in the fight against competing bacteria. Furthermore, nanotechnologies offer a promising area in the prevention of bacterial resistance [67] coupled with an improvement of the oxidative and inflammatory profile of semen contaminated with bacteria.

Finally, semen-processing protocols employing gradient separation techniques based on Percoll [68] or Accudenz [69] have been shown to effectively reduce the amount bacteria in poultry semen and subsequently decrease the necessity for antibiotics in semen extenders directed against these microorganisms.

## 5. Conclusions

As the industrial production of turkeys relies on artificial insemination, it is extremely important to pay attention to the quality of semen intended for assisted reproduction. Bacteriospermia is a common phenomenon occurring in turkey ejaculates. Our analysis revealed that the sperm quality parameters were significantly affected by both the bacterial load and diversity. The sperm motility, membrane integrity, and mitochondrial function are amongst the vitality markers that were mostly affected by the bacterial presence. At the same time, we observed a significant involvement of oxidative stress and inflammatory molecules in the bacteria-inflicted sperm damage. An important observation lied in the role of natural antibacterial proteins, such as cathelicidin and β-defensin in the maintenance of the sperm survival. Our study emphasizes on the criticality of bacteriospermia in turkey breeding and highlights the need to include a microbiological screening of semen samples designated for artificial insemination. Since such currently available microbiological techniques are time-consuming and impractical for the industry, it is necessary to search for reliable biomarkers of bacteriospermia that would allow their use in the form of rapid tests.

## Figures and Tables

**Table 1:** Resistance profiles of bacteria found in turkey semen.

Isolate	Antibiotic	S	I	R
*Bacillus cereus*	C	ND	ND	ND
	LZD	ND	ND	ND
	TGC	ND	ND	ND
*Bacillus subtilis*	C	ND	ND	ND
	LZD	ND	ND	ND
	TGC	ND	ND	ND
*Citrobacter braakii*	ETP	100%	0%	0%
	FEP	100%	0%	0%
	NOR	100%	0%	0%
*Empedobacter brevis*	ETP	ND	ND	ND
	FEP	ND	ND	ND
	NOR	ND	ND	ND
*Enterococcus faecium*	IMP	0%	0%	100%
	LZD	100%	0%	0%
	TGC	100%	0%	0%
*Escherichia coli*	ETP	0%	0%	100%
	FEP	100%	0%	0%
	NOR	100%	0%	0%
*Klebsiella pneumoniae*	ETP	0%	0%	100%
	FEP	100%	0%	0%
	NOR	100%	0%	0%
*Morganella morganii*	ETP	0%	0%	100%
	FEP	100%	0%	0%
	NOR	100%	0%	0%
*Myroides odoratimimus*	C	ND	ND	ND
	LZD	ND	ND	ND
	TGC	ND	ND	ND
*Proteus hauseri*	ETP	100%	0%	0%
	FEP	100%	0%	0%
	NOR	100%	0%	0%
*Proteus mirabilis*	ETP	100%	0%	0%
	FEP	100%	0%	0%
	NOR	100%	0%	0%
*Proteus penneri*	ETP	100%	0%	0%
	FEP	100%	0%	0%
	NOR	100%	0%	0%
*Proteus vulgaris*	ETP	100%	0%	0%
	FEP	100%	0%	0%
	NOR	100%	0%	0%
*Staphylococcus chromogenes*	C	0%	0%	100%
	LZD	100%	0%	0%
	TGC	0%	0%	100%
*Staphylococcus lentus*	C	0%	0%	100%
	LZD	0%	0%	100%
	TGC	0%	0%	100%
*Streptococcus alactolyticus*	C	50%	0%	50%
	LZD	0%	0%	100%
	TGC	0%	0%	100%
*Vagococcus fluvialis*	ETP	0%	0%	100%
	FEP	100%	0%	0%
	NOR	0%	0%	100%

Cefepime (FEP), ertapenem (ETP), chloramphenicol (C), linezolid (LZD), imipenem (IMP), norfloxacin (NOR), tigecycline (TGC), not defined (ND), sensitive (S), intermediate (I), resistant (R).

**Table 2:** Mean values for the qualitative parameters assessed in turkey ejaculates (n = 60).

Parameter	Value (Mean ± S.D.)
Sperm motility (%)	61.61 ± 3.01
Membrane integrity (%)	84.10 ± 1.56
Acrosome integrity (%)	90.77 ± 0.83
Mitochondrial membrane potential (green/red ratio)	0.69 ± 0.02
DNA fragmentation (%)	7.64 ± 0.36
Necrotic cells (%)	3.99 ± 0.29
Concentration of leukocytes (×10^6^/mL)	4.42 ± 1.29
Reactive oxygen species (ROS) production (RLU/s/10^6^ cells)	4.10 ± 0.41
Total antioxidant capacity (μmol Trolox equivalent/g prot)	13.34 ± 1.07
Protein oxidation (nmol PC/mg prot)	2.67 ± 0.56
Lipid peroxidation (µmol MDA/g prot)	0.75 ± 0.09
C-reactive protein (mg/g prot)	0.79 ± 0.05
Interleukin-6 (pg/mg prot)	242.70 ± 11.45
Interleukin-1 (pg/mg prot)	0.09 ± 0.01
Cathelicidin concentration (pg/mg prot)	0.99 ± 0.19
Defensin concentration (µg/mg prot)	0.26 ± 0.04
Bacterial colonies (log CFU/mL)	14.00 ± 1.22

**Table 3:** Correlations amongst selected semen quality parameters, oxidative, and immunological markers of turkey ejaculates (n = 60).

	MOT	MI	AI	ΔΨm	DNA	NC	LEU	ROS	TAC	PC	MDA	CRP	IL-1	IL-6	CATH	DEF	CFU
**MOT**	1	0.665 ***	0.518 **	0.604 ***	−0.544 **	−0.527 **	−0.787 ***	−0.758 ***	0.466 **	−0.543 **	−0.617 ***	−0.467 **	−0.614 ***	−0.557 ***	0.625 ***	0.706 ***	−0.754 ***
**MI**		1	0.383 *	0.583 **	−0.170	−0.527 **	−0.680 ***	−0.529 **	0.470 *	−0.456 *	−0.644 ***	−0.176	−0.304	−0.309	0.541 **	0.626 ***	−0.467 *
**AI**			1	0.208	−0.430 *	−0.396 *	−0.505 **	−0.469 *	0.390 *	−0.427 *	−0.528 **	−0.252	−0.414 *	−0.427 *	0.313	0.348 *	−0.609 **
**ΔΨm**				1	−0.636 ***	−0.633 ***	−0.646 ***	−0.559 ***	0.345 *	−0.412 *	−0.585 ***	−0.349 *	−0.377 *	−0.384 *	0.307	0.477 *	−0.480 **
**DNA**					1	0.592 ***	0.498 **	0.535 **	−0.360 *	0.355 *	0.539 **	0.358 *	0.349 *	0.362 *	−0.405 *	−0.380 *	0.565 ***
**NC**						1	0.487 **	0.490 **	−0.306	0.424 *	0.423 *	0.372 *	0.611 ***	0.696 ***	−0.313	−0.452 *	0.439 *
**LEU**							1	0.668 ***	−0.446 *	0.424 *	0.624 ***	0.409 *	0.503 **	0.509 **	−0.485 **	−0.504 **	0.683 ***
**ROS**								1	−0.620 ***	0.484 *	0.467 *	0.485 *	0.448 *	0.542 **	−0.458 **	−0.464 **	0.668 ***
**TAC**									1	−0.391 *	−0.338 *	−0.112	−0.296	−0.325	0.690 ***	0.545 ***	−0.409 *
**PC**										1	0.646 ***	0.035	0.253	0.304	−0.132	−0.154	0.658 ***
**MDA**											1	0.512 **	0.388 *	0.435 *	−0.389 *	−0.370 *	0.553 **
**CRP**												1	0.516 **	0.566 **	−0.403 *	−0.377 *	0.681 ***
**IL-1**													1	0.779 ****	−0.356 *	−0.388 *	0.559 ***
**IL-6**														1	−0.324 *	−0.365 *	0.513 **
**CATH**															1	0.829 ****	−0.474 **
**DEF**																1	−0.523 **
**CFU**																	1

The interpretation of the results was based on the value of the Pearson’s correlation coefficient: ±0.111–±0.333: weak correlation; ±0.334–±0.666: moderate correlation; ±0.667–±0.999: strong correlation. * *p* < 0.05; ** *p* < 0.01; *** *p* < 0.001; **** *p* < 0.0001. MOT: spermatozoa motility (%); MI: membrane integrity (%); AI: acrosome integrity (%); ΔΨm: mitochondrial membrane potential (JC-1 units); DNA: sperm DNA fragmentation (%); NC: necrotic cells (%); LEU: concentration of leukocytes (×10^6^/mL); ROS: reactive oxygen species production (RLU/s/10^6^ cells); TAC: total antioxidant species (μmol Trolox equivalent/g prot); PC: protein carbonyls content (protein oxidation) (nmol PC/mg prot); MDA: malondialdehyde concentration (lipid peroxidation) (µmol MDA/g prot); CRP: C-reactive protein (mg/g prot); IL: interleukins (pg/mg prot); CATH: cathelicidin concentration (pg/mg prot); DEF: defensin concentration (µg/mg prot); CFU: colony-forming units (log CFU/mL).

**Table 4:** Comparative analysis of the quality groups.

Groups	Excellent (MOT > 70%)	Good (MOT > 50%)	Low (MOT < 50%)
	(n = 22)	(n = 20)	(n = 18)
Sperm motility	77.09 ± 1.02	61.50 ± 1.55 ****^A^	33.50 ± 3.95 ****^B;^ ****^C^
Membrane integrity	91.55 ± 2.80	82.36 ± 1.58 **^A^	74.50 ± 4.20 ****^B;^ *^C^
Acrosome integrity	93.00 ± 2.72	91.29 ± 3.93	85.50 ± 2.27 **^B;^ *^C^
ΔΨm	0.78 ± 0.07	0.68 ± 0.08 **^A^	0.59 ± 0.06 ***^B^
DNA damage	6.55 ± 0.50	7.78 ± 0.50	9.30 ± 0.66 *^B^
Necrotic cells	2.97 ± 0.38	4.16 ± 0.38	5.45 ± 0.58 **^B^
Leukocytes	0.82 ± 0.43	5.17 ± 1.43 ****^A^	9.25 ± 0.79 ****^B;^ ****^C^
ROS production	2.66 ± 0.36	3.85 ± 0.54	7.34 ± 0.52 ****^B;^ ***^C^
TAC	16.43 ± 2.44	13.03 ± 0.81	8.41 ± 1.18 *^B^
Protein oxidation	1.53 ± 0.46	1.88 ± 0.46	6.77 ± 1.92 ***^B;^ **^C^
LPO	0.48 ± 0.08	0.72 ± 0.08	1.33 ± 0.29 ***^B;^ *^C^
CRP	0.71 ± 0.04	0.78 ± 0.04	0.98 ± 0.21
IL-6	0.07 ± 0.01	0.08 ± 0.01	0.14 ± 0.02 ***^B;^ **^C^
IL-1	105.50 ± 11.21	187.60 ± 17.21	622.90 ± 64.00 ***^B;^ ***^C^
CATH	1.96 ± 0.36	0.55 ± 0.12 ****^A^	0.24 ± 0.04 ****^B^
DEF	0.49 ± 0.05	0.13 ± 0.01 ****^A^	0.10 ± 0.01 ****^B^
Bacterial colonies	8.85 ± 2.11	14.19 ± 2.10 *^EA^	23.03 ± 4.08 ****^B;^ **^C^
Bacterial species (sample positivity)	*B. subtilis* (27%)	*B. cereus* (50%)	*B. cereus* (45%)
	*E. brevis* (37%)	*B. subtilis* (50%)	*B. subtilis* (56%)
	*M. odoratimimus* (37%)	*M. morganii* (30%)	*C. braaki* (34%)
	*S. chromogenes* (27%)	*M. odoratimimus* (30%)	*E. coli* (67%)
	*S. alactolyticus* (45%)	*P. hauseri* (40%)	*E. faecium* (56%)
	*M. morganii* (45%)	*P. penneri* (34%)	*K. pneumoniae* (45%)
		*P. vulgaris* (40%)	*P. hauseri* (23%)
		*S. chromogenes* (30%)	*P. mirabilis* (23%)
			*P. penneri* (50%)
			*P. vulgaris* (45%)
			*S. chromogenes* (56%)
			*S. lentus* (45%)
			*V. fluvialis* (45%)

^A^ EX versus GO; ^B^ EX versus LO; ^C^ GO versus LO. * *p* < 0.05; ** *p* < 0.01; *** *p* < 0.001; **** *p* < 0.0001. Units: sperm motility (%); membrane integrity (%); acrosome integrity (%); ΔΨm (mitochondrial membrane potential) (JC-1 units); DNA fragmentation (%); necrotic cells (%); leukocytes (×10^6^/mL); ROS (reactive oxygen species) production (RLU/s/10^6^ cells); TAC (total antioxidant capacity) (μmol Trolox equivalent/g prot); protein oxidation (nmol PC/mg prot); LPO (lipid peroxidation) (µmol MDA/g prot); CRP (C-reactive protein) (mg/g prot); IL-6 (Interleukin-6) (pg/mg prot); IL-1 (Interleukin-1) (pg/mg prot); CATH (Cathelicidin) (pg/mg prot); DEF (β-Defensin) (µg/mg prot); bacterial colonies (log CFU/mL).

## Data Availability

The data presented in this study are available on request from the corresponding author.
